# Caging the Beast: TRIM5α Binding to the HIV-1 Core

**DOI:** 10.3390/v3050423

**Published:** 2011-04-27

**Authors:** Felipe Diaz-Griffero

**Affiliations:** Department of Microbiology and Immunology, Albert Einstein College of Medicine, Bronx, NY 10461, USA; E-Mail: Felipe.Diaz-Griffero@einstein.yu.edu; Tel.: +1-(718)-678-1191; Fax: +1-(718)-632-4338

**Keywords:** HIV-1, TRIM5α, capsid, binding, core

## Abstract

The potent HIV-1 inhibitor TRIM5α blocks HIV-1 infection by accelerating the uncoating of HIV-1. TRIM5α is known to form higher-order self-association complexes that contribute to the avidity of TRIM5α for the HIV-1 capsid, and are essential to inhibit infection; these higher-order self-association complexes are dependent upon an intact B-box 2 domain. Even though the ability to form higher-order self-association complexes resembles the clathrin triskelion that forms a protein array, or cage, around the endocytic vesicle, evidence for the ability of TRIM5α to assemble a similar type of structure surrounding the HIV-1 core has been lacking. Recent work by Ganser-Pornillos, Chandrasekaran and colleagues has now demonstrated the ability of the restriction factor TRIM5α to “cage” or “net” the HIV-1 core by forming an hexagonal array on the surface of the viral capsid [1]. This hexagonal array is strikingly similar in design to the array formed by the clathrin triskelion on the surface of the clathrin-coated endocytic vesicle. This remarkable finding represents an important advance on our understanding of the restriction factor TRIM5α, and suggests that TRIM5α cages the HIV-1 core in order to terminate infection. The present note discusses the implications of this discovery.

“Caging the beast”, *i.e.*, putting the HIV-1 core in a cage, seems to be the solution to all our problems. Even though the battle with HIV-1 is far from over, Ganser-Pornillos and colleagues, in what could be considered a remarkable achievement in the HIV-1 field, demonstrated that the potent HIV-1 inhibitor TRIM5α cages the HIV-1 core in order to terminate infection [1].

The rhesus macaque endogenously expressed protein tripartite motif 5α (TRIM5α_rh_) potently blocks HIV-1 infection [2]; the restriction of HIV-1 by TRIM5α_rh_ is 100–1000 fold [3,4]. Because TRIM5α is a natural mechanism to block retroviruses such as HIV-1, researchers have tried to unlock the mystery behind the protein for many years now. Deciphering this mechanism could be a “Rosetta stone” for creating alternative treatments against HIV-1/AIDS.

TRIM5α_rh_ comprises four domains, including the RING, B-box 2, coiled-coil and B30.2/SPRY (Figure 1A). The RING domain exhibits E3 ubiquitin ligase activity [5–9], and the contribution of this activity to the ability of TRIM5α to restrict HIV-1 is under intense investigation. The B-box 2 domain contributes to the avidity of TRIM5α_rh_ for the HIV-1 capsid [10–12]; the avidity of TRIM5α_rh_ for capsid is improved by TRIM5α inter-dimer interactions (Figure 1A). The coiled-coil allows TRIM5α dimer formation [8,9]. Lastly, the B30.2/SPRY domain of TRIM5α provides the specificity for capsid recognition [13,14]. All four of these domains are important for HIV-1 restriction.

Since the discovery of TRIM5α by Matthew Stremlau [2], the field recognized the aggregation/assembly properties of the protein. Early studies demonstrated that mutations in the B-box 2 domain of TRIM5α, such as R121E, were apparently less prone to cause aggregation in the cytoplasm [10]; however, TRIM5α bearing this particular mutation did not restrict HIV-1 [10]. In quantitative capsid-binding assays, B-box 2 domain mutants, such as R121E, had a range of binding affinities to the HIV-1 capsid that were directly correlated with HIV-1 restriction [12]. Interestingly, TRIM5α B-box 2 domain mutants are less prone to aggregate. Perhaps counter-intuitively, the field began to consider aggregation as an important factor for the ability of TRIM5α to restrict HIV-1 infection. This idea led to experiments that tested the ability of TRIM5α to self-associate into higher-order structures, as had been shown for the protein clathrin [15–17]. According to this simple model, one TRIM5α dimer associates with another (Figure 1A) [11,12], and these higher-order structures directly impact the binding affinity of TRIM5α_rh_ for the HIV-1 capsid. The fact that TRIM5α_rh_ was able to undergo higher-order self-association suggested that TRIM5α_rh_ could potentially form a lattice or an array on top of the capsid, just like the clathrin triskelion in clathrin coated vesicles; however, there was not at that point any direct evidence to support this hypothesis for TRIM5α. Ganser-Pornillos and colleagues undertook studies to physically visualize the binding of TRIM5α to the HIV-1 capsid; they demonstrated that TRIM5α proteins form an hexagonal array on top of the pre-assembled arrays of the HIV-1 capsid protein. Although the TRIM5α hexameric array had a striking resemblance to the clathrin-coated vesicle seen by electron microscopy, it is important to mention that the final outcome of both processes is different. This remarkable observation provided new insights on the mechanism used by TRIM5α_rh_ to block HIV-1.

By using negative-staining electron microscopy of the TRIM5-21R protein, a TRIM5α_rh_ protein containing the RING domain of human TRIM21 [6], the authors visualized for the first time the ability of TRIM5-21R to spontaneously form two-dimensional paracrystalline hexagonal arrays [1]. Although these hexagonal arrays are formed at relatively low concentrations of protein (∼10 μM), the SPRY domain of TRIM5α_rh_ was not required for array assembly. Thus the RING, Bbox-2 and coiled-coil domains mediate the assembly of hexagonal arrays, where as the SPRY domain might be dictating where these hexagonal arrays form. The authors went on to confirm that mutants of the B-box-2 domain, such as R121E, have reduced binding to capsid, as a result of impaired higher-order self-association, in agreement with previous biochemical observations [12]. Furthermore, TRIM5-21R bearing the B-box 2 domain mutation R121E failed to form hexagonal arrays, implying that the B-box 2 domain might be involved in key interactions during lattice assembly. The fact that purified TRIM5-21R spontaneously assembles into hexameric arrays in the absence of capsid suggests that this process might be regulated in the cellular environment by post-translational modifications or other factors [8,9]. As expected, the formation of hexagonal TRIM5α_rh_ arrays was facilitated when planar sheets of the capsid-nucleocapsid protein were added to the mixture.

Taken together, these results have several important implications for viral restriction:

1. According to the model proposed by Ganser-Pornillos and colleagues, the RING domain is localized on the surface of the core when the hexagonal arrays are formed. One possible scenario is that several RING domains could be recruiting E2 enzymes to the surface of the core to perform TRIM5α_rh_ ubiquitylation that will lead to core disassembly (Figure 1B). One could envision a model in which the self-ubiquitylation of TRIM5α is required to remove TRIM5α when forming hexagonal structures on the surface of the capsid [18–20]; removal of TRIM5α from the surface of the capsid will allow a decrease on the amount of particulate capsid during infection, thereby promoting rapid uncoating. In addition, our own unpublished results have suggested that the RING domain could also be playing a role in higher-order self-association, as shown for the B-box 2 domain. Indeed, RING domains have been shown to form higher-order interactions in other E3 enzymes [21–23].
2. The B-box 2 domain is likely to be the anchor in the array, since Ganser-Pornillos and colleagues demonstrate that the B-box-2 domain mutation R121E does not form hexagonal arrays. This observation agrees with previous results demonstrating that the B-box 2 domain is important for higher-order self-association (Figure 1A), which increases the avidity of TRIM5α_rh_ for the HIV-1 capsid [12].
3. The coiled-coil allows formation of the TRIM5α_rh_ dimer (Figure 1A) that is likely to be the minimal unit required for assembly of the hexagonal array, in analogy to the triskelion building block of the clathrin lattice [15–17].
4. The SPRY domain is likely to be in direct contact with capsid, as suggested by biochemical analyses from several laboratories. Thus the SPRY domain can provide specificity that dictates where this array will nucleate. The fact that the SPRY domain is not required for lattice formation opens the possibility that proteins related to TRIM5α_rh_ such as TRIM6, TRIM21 and TRIM34 are recruited to the array. Recruitment of other TRIM proteins to the array might be important for the fate of the HIV-1 core following array formation; therefore, it might be important to evaluate the fate of the capsid in cells containing endogenously expressed TRIM5α_rh_ where expression of the different TRIM5-related proteins such as TRIM6, TRIM34 and TRIM21 are knocked down. We have observed acceleration of uncoating only in cells over expressing TRIM5α_rh_ raising the possibility that this could be different in a scenario where different TRIMs are recruited to the core. This type of mixed array will make difficult the study of the contribution of TRIM5α domains that contain enzymatic activity to restriction, such as the RING domain, because of function complementation by the same domains from other TRIM proteins in the array.

It is well-established biochemically that the presence of TRIM5α_rh_ during HIV-1 infection correlates with a decrease of particulate capsid, or what has been called “rapid uncoating/disassembly of the HIV-1 core” [24–26]. However, when comparing HIV-1 core disassembly to the disassembly of the clathrin coat [15–17], the first thing that comes to mind is the source of energy for this event. Whether the energy required for uncoating post-TRIM5α_rh_ binding is derived from the removal of self-ubiquitylated TRIM5α_rh_ proteins from the surface of the core or an independent source, it is likely that this process requires energy. Future experiments will determine the preceding steps to “caging” of the core to fully understand the restriction mechanism used by TRIM5α_rh_.

## Figures and Tables

**Figure 1:**
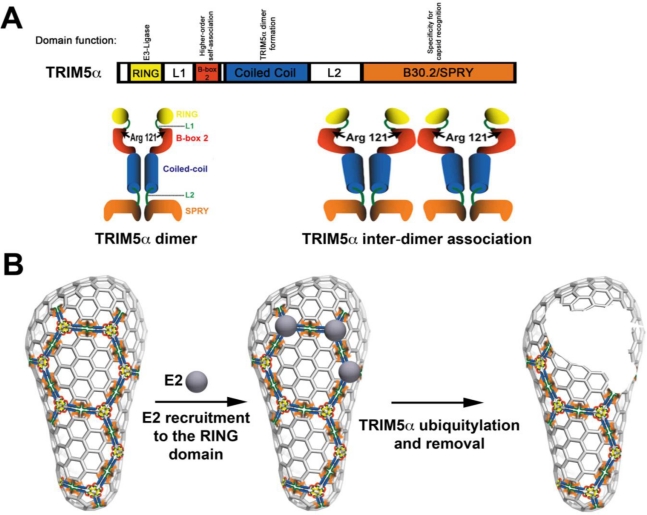
Current model for acceleration of uncoating mediated by TRIM5α. (**A**) The linear box diagram represents the different domains of the TRIM5α protein with their respective known functions. TRIM5α forms a dimer, which is stabilized by the coiled-coil domain. In addition, TRIM5α forms higher-order self-association complexes (TRIM5α inter-dimer association) that are dependent upon an intact B-box 2 domain. (**B**) According to the model proposed by Ganser-Pornillos and colleagues, TRIM5α initially cages the core. Because the RING domain is exposed on the surface of the core, we can envision the recruitment of E2 enzymes that will ubiquitylate TRIM5α. Removal of ubiquitylated TRIM5α, for subsequent degradation, from the surface of the core will damage the core triggering the occurrence of premature uncoating.
